# Assessment of cardiac autonomic dysfunction in patients with chronic kidney disease using heart rate variability

**DOI:** 10.6026/973206300212069

**Published:** 2025-07-31

**Authors:** Devpriya Shukla, Amit Saxena, Anju Jha, Raghav Gupta

**Affiliations:** 1 Department of General Medicine, Bundelkhand Medical College, Sagar, Madhya Pradesh, India; 2 Department of Physiology, Bundelkhand Medical College, Sagar, Madhya Pradesh, India; 3 Department of Dermatology, Bundelkhand Medical College, Sagar, Madhya Pradesh, India

**Keywords:** cardiac autonomic dysfunction, chronic kidney disease, heart rate variability, baroreflex sensitivity

## Abstract

Cardiac autonomic dysfunction has been majorly linked with chronic kidney disease (CKD) and has related this condition to an increase in cardiovascular risk. This study compared heart rate variability (HRV) and baroreflex sensitivity among 120 patients with CKD and 60 healthy individuals. Patients with CKD exhibited a significant decrease in the HRV parameters and baroreflex sensitivity (p < 0.001). The more advanced the CKD stage and the worse the eGFR, the worse the autonomic dysfunction. A non-invasive measure referred to as HRV analysis can be conducted to stratify early cardiovascular risks in CKD.

## Background:

Chronic kidney disease is a significant health burden in the world, with an estimated about 1015 percent of the adults in the world population being affected [1]. CKD is defined by a gradual decline in the functioning of the kidneys as time goes on, which consequently results in several complications, such as cardiovascular disease, which has been the most dominant cause of morbidity and mortality in this group of patients [2]. Cardiovascular mortality rate is significantly higher in CKD patients compared with the general population and patients in end stages are shown to have a 10-20 times higher risk of dying due to CVD. This has led to a syndrome known as cardiac autonomic dysfunction [3]. The autonomic nervous system performs a crucial role in cardiovascular homeostasis by controlling heart rate, blood pressure and vascular tone and any alteration of this homeostasis could give rise to increased exposure to arrhythmias, sudden cardiac death, intradialytic hypotension and heart failure [4]. Sympathetic overactivation takes place early in the course of the disease and is directly related to the degree of renal dysfunction. At the same time, the parasympathetic system increasingly loses its abilities so that the inhibitory effects of the vagus on the sinoatrial node are reduced [5]. Other causative factors are a compromised baroreceptor response, reduced chemoreceptor sensitivity, stimulation of the renin-angiotensin-aldosterone system and cardiovascular structural remodelling. The analysis of heart rate variability serves as a valuable indicator of cardiac autonomic status and is a non-invasive measurement [6]. Heart Rate Variability (HRV) serves as a metric for the interplay between sympathetic and parasympathetic afferents to the heart and is quantitatively assessed [7]. The time-domain HRV measures, namely SDNN and RMSSD, indicate overall HRV and sympathetic activity, respectively, while frequency-domain analysis elucidates the sympathetic and parasympathetic components of heart rate control [8]. New research findings revealed changes in the HRV pattern of CKD patients that being a decreased total variability and parasympathetic dysfunction [9]. The extent of HRV impairment seems to be linked with the severity of CKD and can be prognostic relative to cardiovascular events [10]. However, the overall evaluation of cardiac autonomic dysfunction with various parameters of HRV at various stages of CKD is not abundant. Another key product of autonomic assessment, baroreflex sensitivity, measures the capacity of the cardiovascular system to restore the blood pressure to a steady state by altering the heart rate and vascular tone in the process of reflex [11]. Significant disturbance of the baroreflex ability has been reported in patients with CKD and this leads to the instability of blood pressure and cardiovascular acuity [12]. The correlation between the parameters of HRV and common cardiovascular risk factors of patients with CKD needs to be clarified further. Therefore, it is of interest to evaluate cardiac autonomic dysfunction in patients with CKD, through analysis of HRV and measurement of the baroreflex sensitivity, to establish the connection between the parameters of autonomic neuropathy and the degree of CKD and to raise the possibility of predictors of cardiovascular risk stratification in a group of high-risk patients.

## Materials and Methods:

The study included 120 patients with CKD stages 3-5 (not on dialysis) and 60 age- and sex-matched healthy controls. CKD patients were recruited from the outpatient Medicine OPD, while healthy controls were recruited from the hospital staff and community volunteers.

## Inclusion criteria:

CKD patients were included if they were aged 18-75 years with stable CKD stages 3-5 (estimated glomerular filtration rate 15-59 mL/min/1.73m^2^) for at least 3 months.

## Exclusion criteria:

Active cardiovascular disease (myocardial infarction, unstable angina or stroke within 6 months), cardiac arrhythmias, pacemaker implantation, severe heart failure (NYHA class III-IV) and uncontrolled diabetes mellitus (HbA1c >9%), current use of antiarrhythmic medications, pregnancy and inability to perform autonomic function tests. Healthy controls were aged 18-75 years with normal kidney function (eGFR >90 mL/min/1.73m^2^), no history of cardiovascular disease, diabetes mellitus, or hypertension and not taking any medications affecting cardiovascular function.

## Clinical assessment:

All participants underwent a comprehensive clinical evaluation, including medical history, physical examination and laboratory investigations. Blood samples were collected after 12-hour fasting for measurement of serum creatinine, urea, electrolytes, hemoglobin, albumin, calcium, phosphorus and parathyroid hormone. The estimated glomerular filtration rate was calculated using the CKD-EPI equation. Blood pressure was measured using a standardized protocol with participants in the sitting position after 5 minutes of rest.

## Heart rate variability analysis:

ECG recordings were performed in a quiet, temperature-controlled room between 9:00 AM and 12:00 PM to minimize circadian variations. Participants were instructed to avoid caffeine, alcohol and tobacco for 24 hours before testing and to maintain their regular medications. After 10 minutes of supine rest, continuous ECG was recorded for 5 minutes using a 12-lead ECG system (CardioNet Pro, MedTech Systems) with a sampling rate of 1000 Hz. Participants were instructed to breathe normally and remain still during the recording. ECG signals were digitally filtered and manually reviewed to exclude ectopic beats and artifacts.

HRV analysis was performed using specialized software (HRV Analysis Pro v2.1). Time-domain parameters calculated included:

[1] SDNN: standard deviation of all NN intervals (ms)

[2] RMSSD: root mean square of successive differences between NN intervals (ms)

[3] pNN50: percentage of successive NN intervals differing by >50 ms (%)

Frequency-domain analysis was performed using the Fast Fourier Transform with the following frequency bands:

[1] Low frequency (LF): 0.04-0.15 Hz (ms^2^)

[2] High frequency (HF): 0.15-0.40 Hz (ms^2^)

[3] LF/HF ratio: marker of sympathovagal balance

## Baroreflex sensitivity assessment:

Baroreflex sensitivity was evaluated by the Valsalva maneuver conducted in the supine position. Participants were directed to exhale into a mouthpiece linked to a pressure manometer, sustaining 40 mmHg for 15 seconds. Continuous electrocardiogram and non-invasive blood pressure monitoring were conducted during the examination. Baroreflex sensitivity was determined as the slope of the regression line correlating changes in RR interval with alterations in systolic blood pressure during phase II of the Valsalva maneuver.

## Statistical analysis:

Statistical analysis was conducted utilizing SPSS version 26.0. Continuous variables were assessed for normalcy with the Shapiro-Wilk test. Normally distributed variables were presented as mean ± standard deviation, whereas non-normally distributed variables were presented as median (interquartile range). Categorical variables were represented as frequencies and percentages. Comparisons between chronic kidney disease patients and controls were conducted utilizing Student's t-test for regularly distributed variables and the Mann-Whitney U test for non-normally distributed variables. The chi-square test was employed for categorical variables. One-way ANOVA accompanied by post-hoc Tukey's test was employed to compare HRV values across various stages of CKD. Correlation analysis utilized Pearson's correlation coefficient for regularly distributed variables and Spearman's correlation coefficient for non-normally distributed variables. Multiple linear regression analysis was employed to ascertain independent determinants of HRV parameters. A p-value less than 0.05 were deemed statistically significant. Power analysis demonstrated that a sample size of 120 chronic kidney disease patients and 60 controls would have 80% power to identify a 20% disparity in heart rate variability parameters between the groups, given an alpha level of 0.05

## Results:

The study included 120 CKD patients (mean age 58.4 ± 12.8 years, 67% male) and 60 healthy controls (mean age 56.1 ± 11.4 years, 63% male). There were no significant differences in age, sex, or body mass index between groups (p > 0.05). Among CKD patients, 45 (37.5%) had stage 3a CKD, 35 (29.2%) had stage 3b, 25 (20.8%) had stage 4 and 15 (12.5%) had stage 5. The mean eGFR was 38.6 ± 15.2 mL/min/1.73m^2^ in CKD patients versus 96.4 ± 12.8 mL/min/1.73m^2^ in controls (p < 0.001). Hypertension was present in 89 (74.2%) CKD patients have diabetes mellitus in 52 (43.3%) and cardiovascular disease in 28 (23.3%). CKD patients had significantly higher systolic blood pressure (142.6 ± 18.4 vs 118.3 ± 12.6 mmHg, p < 0.001), serum creatinine (2.8 ± 1.4 vs 0.9 ± 0.2 mg/dL, p < 0.001) and lower hemoglobin levels (10.8 ± 1.9 vs 13.6 ± 1.4 g/dL, p < 0.001) compared to controls. CKD patients demonstrated significantly impaired HRV parameters compared to healthy controls across all measured indices. Time-domain analysis revealed marked reductions in overall HRV and parasympathetic activity. SDNN was significantly lower in CKD patients (28.4 ± 12.6 ms) compared to controls (45.8 ± 18.2 ms, p < 0.001), representing a 38% reduction. RMSSD, a marker of parasympathetic activity, was similarly reduced (22.1 ± 10.8 ms vs 38.6 ± 15.4 ms, p < 0.001), indicating a 43% decrease. The percentage of successive NN intervals differing by >50 ms (pNN50) was also significantly lower in CKD patients (8.4 ± 6.7% vs 18.2 ± 11.3%, p < 0.001). Frequency-domain analysis revealed profound alterations in autonomic balance. High-frequency power, reflecting parasympathetic activity, was markedly reduced in CKD patients (142.3 ± 89.7 ms^2^ vs 284.6 ± 156.3 ms^2^, p < 0.001), representing a 50% decrease. Low-frequency power was also reduced but to a lesser extent (186.4 ± 112.8 ms^2^ vs 241.7 ± 134.5 ms^2^, p = 0.008). Consequently, the LF/HF ratio was significantly elevated in CKD patients (2.8 ± 1.4 vs 1.6 ± 0.7, p < 0.001), indicating sympathetic predominance. Baroreflex sensitivity was significantly impaired in CKD patients compared to controls (8.2 ± 4.1 ms/mmHg vs 15.7 ± 6.8 ms/mmHg, p < 0.001), representing a 48% reduction. This impairment was observed across all CKD stages, with progressive worsening as kidney function declined. Analysis of HRV parameters across different CKD stages revealed progressive deterioration with advancing disease severity. Patients with stage 3a CKD showed mild but significant reductions in HRV parameters compared to controls, while those with stages 4-5 demonstrated more pronounced abnormalities. SDNN decreased progressively from 34.2 ± 11.8 ms in stage 3a to 18.6 ± 8.4 ms in stage 5 (p < 0.001 for trend). Similarly, RMSSD declined from 26.8 ± 9.6 ms in stage 3a to 14.2 ± 6.8 ms in stage 5 (p < 0.001 for trend). HF power showed the most dramatic reduction, decreasing from 184.5 ± 78.3 ms^2^ in stage 3a to 76.8 ± 42.1 ms^2^ in stage 5 (p < 0.001 for trend). The LF/HF ratio increased progressively with advancing CKD stage, from 2.1 ± 0.9 in stage 3a to 4.2 ± 1.8 in stage 5 (p < 0.001 for trend), indicating increasing sympathetic predominance with disease progression. Strong inverse correlations were observed between eGFR and most HRV parameters. SDNN correlated positively with eGFR (r = 0.68, p < 0.001), as did RMSSD (r = 0.71, p < 0.001) and HF power (r = 0.74, p < 0.001). The LF/HF ratio showed a strong negative correlation with eGFR (r = -0.69, p < 0.001). Baroreflex sensitivity also correlated positively with eGFR (r = 0.63, p < 0.001). Hemoglobin levels showed modest positive correlations with HRV parameters (r = 0.32-0.45, p < 0.01), while systolic blood pressure correlated negatively with RMSSD (r = -0.38, p < 0.001) and HF power (r = -0.41, p < 0.001). Multiple linear regression analysis identified eGFR as the strongest independent predictor of HRV parameters, accounting for 42-58% of the variance in different measures. Age, hemoglobin level and systolic blood pressure were additional significant predictors, but their contributions were modest compared to eGFR. For SDNN, the regression model (R^2^ = 0.72, p < 0.001) included eGFR (β = 0.58, p < 0.001), age (β = -0.24, p < 0.001) and hemoglobin (β = 0.18, p = 0.002) as significant predictors. Similar patterns were observed for other HRV parameters (Table 1-5 - see PDF).

## Discussion:

This study provides comprehensive evidence of significant cardiac autonomic dysfunction in CKD patients, as demonstrated by markedly reduced HRV parameters and impaired baroreflex sensitivity across all disease stages. Our findings confirm and extend previous observations regarding the prevalence and severity of autonomic dysfunction in this high-risk population [13]. The observed 38-50% reduction in HRV parameters in CKD patients compared to healthy controls is consistent with prior studies demonstrating autonomic impairment in chronic kidney disease [9]. The magnitude of these changes is clinically significant, as reduced HRV has been associated with increased cardiovascular mortality in various patient populations [10]. Our results align with the findings of Clyne *et al.*, who reported significant correlations between HRV parameters and estimated glomerular filtration rate in CKD patients [14]. The progressive deterioration of autonomic function with advancing CKD stage observed in our study supports the concept that autonomic dysfunction is not merely a consequence of end-stage renal disease but occurs early in the disease course and worsens with declining kidney function. This finding has important clinical implications, as it suggests that autonomic assessment may serve as a marker of disease progression and cardiovascular risk stratification even in earlier CKD stages [15]. The predominant pattern of autonomic dysfunction observed was characterized by reduced parasympathetic activity, as evidenced by decreased RMSSD and HF power, combined with sympathetic predominance reflected by elevated LF/HF ratios [5]. This pattern is consistent with the established pathophysiology of CKD, which involves early sympathetic activation due to factors such as arterial stiffening, volume overload and activation of the renin-angiotensin-aldosterone system [16]. The parasympathetic dysfunction likely reflects structural and functional changes in cardiac innervation associated with chronic inflammation and uremic toxicity. Our findings of significantly impaired baroreflex sensitivity in CKD patients corroborate previous research demonstrating altered baroreceptor function in this population [11]. The 48% reduction in baroreflex sensitivity observed in our study is comparable to that reported in other chronic disease states associated with increased cardiovascular risk [12]. Impaired baroreflex function contributes to blood pressure variability and reduced cardiovascular adaptability, potentially explaining the increased risk of orthostatic hypotension and intradialytic hypotension commonly observed in CKD patients [17]. The strong correlations between eGFR and autonomic parameters observed in our study suggest that kidney function itself may be a primary determinant of autonomic dysfunction in CKD [18]. This relationship remained significant even after adjusting for traditional cardiovascular risk factors, supporting the concept that uremia and its associated metabolic derangements directly impact autonomic nervous system function [19]. The independent contribution of hemoglobin levels to HRV parameters may reflect the role of anemia in cardiovascular dysfunction in CKD patients.

In a clinical setting, we found that HRV analysis may be a meaningful non-invasive cardiovascular risk assessment tool for CKD patients [20]. This gradual development of autonomic dysfunction as the CKD stage progresses can be used to designate the patients at an early stage of high risk, where they can be closely monitored and treated regarding cardiovascular specialities [4]. Besides, HRV parameters could also be used as surrogate endpoints in assessing the cardiovascular outcomes of the therapeutic interventions in CKD patients. A few mechanisms can be the cause of autonomic dysfunction in CKD individuals [1]. Inflammatory conditions in chronic kidney disease, which are chronic inflammations, may have direct contact with autonomic centers in both the brainstem and peripheral autonomic ganglia [2]. Certain uremic toxins can influence the production and release of neurotransmitters and the imbalances in electrolytes typical in CKD can influence nerve conduction and cardiac excitability [21]. The impairment of the baroreceptor and the autonomic dysfunction can be caused by vascular calcification and arterial stiffening that characterize CKD [11]. Therapies, which could benefit autonomic performance (exercise training, pharmacological modulation of the renin-angiotensin system and control of mineral bone disorders) could help decrease the cardiovascular risk of CKD patients. Using an HRV analysis, therapeutic decisions could be made through regular assessment of autonomic activity and determining response to treatment [20].

## Strengths and limitation s of the study:

The proposed study has a number of strengths, such as a rather large sample size, assessment of various HRV parameters, baroreflex sensitivity testing and systematic study of various CKD stages. The high fidelity of our results is also relevant due to the fact that patients with acute cardiovascular events are excluded and the requirements of standardized recording conditions are observed. Nevertheless, one must remember a number of limitations. The cross-sectional design does not allow for determining the causal relationships and evaluating the changes in autonomic over time. The lack of generalizability can be defined by the fact that the study population was sampled in one center. We failed to evaluate 24-hour HRV parameters that could give us more knowledge about the circadian autonomic patterns. The lack of dialysis patients restricts the applicability of our results to this significant group of CKD diseases. Additional longitudinal research is necessary to determine the prognostic role of HRV parameters in the cardiovascular outcomes of CKD patients and to determine the impacts of a particular intervention on autonomic activity. Studies of new HRV parameters and correlation with cardiovascular risk biomarkers can give further information about the pathophysiology of autonomic dysfunction in CKD.

## Conclusion:

There is a big cardiac autonomic dysfunction in the patients of CKD, which increases with the progression of the disease and is also linked with the deterioration in renal function. HRV analysis creates an option for non-invasive early-stage risk stratification and intervention guidance. The prognostic significance and therapeutic value of HRV in reducing cardiovascular risk showed be analyzed in future studies.
